# Reconstruction of massive tibial defect caused by osteomyelitis using induced membrane followed by trifocal bone transport technique: a retrospective study and our experience

**DOI:** 10.1186/s12893-021-01421-x

**Published:** 2021-12-15

**Authors:** Yimurang Hamiti, Maimaiaili Yushan, Cheng Lu, Aihemaitijiang Yusufu

**Affiliations:** Department of Microrepair and Reconstructive Surgery, The First Affiliated Hospital of Xinjiang Medical University, Urumqi, Xinjiang People’s Republic of China

**Keywords:** External fixator, Induced membrane, Ilizarov technique, Trifocal bone transport, Osteomyelitis

## Abstract

**Objective:**

To evaluate clinical outcomes of the application of induced membrane followed by trifocal bone transport technique in the treatment of massive tibial defect caused by osteomyelitis.

**Method:**

A total of 18 eligible patients with tibial defect > 6 cm caused by osteomyelitis who were admitted to our institution from January 2010 to January 2016 and treated by induced membrane followed by trifocal bone transport technique. There were 12 male and 6 females with an average age of 40.4 years old. A detailed demographic data (age, sex, etiology, previous operation time, defect size and location, interval from Masquelet technique to trifocal bone transport technique, external fixation index (EFI), duration of regenerate consolidation and docking union) were collected, bone and functional outcomes were evaluated by Association for the Study and Application of the Method of Ilizarov (ASAMI) scoring system. Complications during and in the period of follow up were recorded and evaluated by Paley classification at a minimum follow-up of 2 years.

**Results:**

The etiology include posttraumatic osteomyelitis in 13 cases and primary osteomyelitis in 5 cases. An average of previous operation time was 3.4 times. Mean tibial defect after radical debridement was 6.8 cm. An average interval duration from formation of induced membrane to trifocal bone transport was 4.8 weeks. An average of EFI was 37.1 days/cm, the duration of regenerate consolidation and docking union were 124.7 days and 186.4 days, respectively. An average time of follow-up after removal of external fixator was 28.5 month without recurrence of osteomyelitis. The bony outcome was excellent in 6 cases, good in 8 cases, fair in 3 cases and poor in 1 case, and functional outcome was excellent in 4 cases, good in 10 cases, fair in 2 cases and poor in 2 cases. The most common complication was pin tract infection which occurred in 15 cases and there were no major complications such as nerve or vascular injury.

**Conclusion:**

Massive tibial defect caused by osteomyelitis can be successfully treated first stage using induced membrane followed by second stage using trifocal bone transport technique, which is an effective method in terms of radical elimination of osteomyelitis with expected clinical outcomes.

## Introduction

Management of tibial defect caused by primary or secondary osteomyelitis is a challengeable troublesome situation, even for skillful and experienced orthopedic and plastic surgeon, especially massive defect (> 6 cm) associated with soft tissue loss which compromised the surrounding envelope of blood supply for bone healing and result in “chaotic” condition requires individualized strategic reconstructive approach to reach expected clinical outcomes [1]. Several treatment options have been proposed and stood the test of time in the reconstruction of long bone defect, current treatment techniques range from autologous bone grafting, Masquelet technique (also known as induced membrane technique), Ilizarov technique based on distraction osteogenesis, free vascularized or non-vascularized fibular grafting, depending on the anatomical location and size of the defect as well as associated injuries [2–10]. Aforementioned treatment approach was characterized by its advantages and shortcomings and there is no consensus on standard treatment protocol to follow while encountered with such devastating situation which is discouraged to both surgeons and patients if unexpected complications have occurred and result in poor treatment outcomes.

Masquelet technique was firstly introduced in 1986 and gained its popularity in the reconstruction of bone defect by separated two stage with satisfied clinical outcomes [11]. Ilizarov bone transport technique based on distraction osteogenesis have been applied in treatment of bone defect, especially in cases associated with soft tissue loss given its naturally advantages of simultaneous histogenesis result in expected treatment outcomes. Combination of Masquelet technique and Ilizarov bone transport (bifocal) technique have been reported and approved its usefulness in the successful reconstruction of bone defect [12–16].

To overcome the inevitable long duration of external fixation time (EFI) using bifocal bone transport technique and reduce its associated complications, studies have been conducted to reduce the EFI such as trifocal (double-level) bone transport technique[1], implantation of plate [17] or intramedullary nailing [18, 19] after removal of external fixator once reached the docking, plate assisted segmental bone transport with motorized lengthening nails and locking plates in the treatment of femoral and tibial defects with successful outcomes[20, 21].

In this study, we presented clinical outcomes in 18 patients with massive tibial defects caused by osteomyelitis who underwent operation using induced membrane followed by trifocal bone transport and to summarize our experiences regarding these combined surgical approaches in the reconstruction of massive tibial defect.

### Patient and method

Patients with massive tibial defect (> 6 cm after radical debridement) caused by osteomyelitis (primary or secondary to trauma) who were treated by the application of the induced membrane technique in the first stage followed by Ilizarov technique using trifocal (double level) bone transport in the second stage between January 2010 to January 2016 were included. Patients were excluded if tibial defect caused by other etiology such as tumor resection or defect more than 6 cm but solely treated with the Induced membrane technique or bone transport technique. Patients who were loss of follow-up or not willing to participate in the present study were also excluded. All enrolled patients were informed and consented about treatment duration and fully aware of the possible associated complications after permission from the Ethics Committee of our institute. All operations were performed by the same surgical team who participated in all surgical procedures and it was a multiple surgeons experience from a single-center study.

### Preoperative preparation

A detailed medical history was obtained include initial injury and previous operation. A thorough examination was performed. Routine radiographs, including anteroposterior and lateral radiographs of the affected limb, and an MRI examination to assess the primary extent of infection, the bone quality, the expected defect after debridement.

### Surgical technique

#### First stage

Patient was placed in a supine or lateral position under general or spinal anesthesia. Radical debridement of infected or devitalized bone and soft tissues was removed until reaching healthy bleeding bone with adequate soft-tissue cover. Any implants or external fixation devices were removed that had been placed previously. The extent of bony resection was at least 0.5 cm away from the infected area. And the cortical bleeding, also named the “paprika sign”, was identified as vital osseous tissue [22]. Samples from the infected area were obtained for bacterial culture and histological analysis to guide the appropriate postoperative antibiotics. Irrigate the wound with hydrogen peroxide solution, 0.9% normal saline, and iodophor saline solution during and after debridement. According to physical and imaging examinations, a suitable external device was installed for the stabilization of the defect area. Measured and filled the bone void by using a polymethylmethacrylate (PMMA) spacer mixed with 10% vancomycin. Frequent irrigation with saline to avoid the “heat” injury to the surrounding tissues caused by spacers. Close the wound adequately in tension free manner by direct closure or use of free/local flap if necessary.

#### Second stage

This stage was performed after the treatment of systemic antibiotics and there was neither local nor systemic inflammation noted which can be evaluated by the trending levels of white blood cell counts, C-reactive protein (CRP) and erythrocyte sedimentation rate (ESR). PMMA spacer was removed after confirmation of the induced membrane formation in an average of 4.8 weeks (ranged 4–7 weeks). Close the biological membrane carefully. Subsequently, two osteotomies were performed percutaneously according to pre-designed locations of the tibia for the trifocal (double-level) bone transport.

### Postoperative management and outcome evaluation

Patients were encouraged for passive knee and ankle joint exercise and gradually weight bearing on postoperative day one. Bone segments transportation was initiated after 7–10 days latency period. Bone transport in the same direction, the fragment near the bone defect was transported at a rate of 0.5 mm and 4 times/day, and another fragment far from the defect was transported 0.25 mm and 4 times/day. For converged bone transport, each fragment on both end of the bone defect proceeded at a rate of 0.25 mm and 4 times/day. The rhythm was adjusted according to the distance and quality of each distracted region. Transport was stopped until the segment reached the docking site and early full-bearing was encouraged. Antibiotics was given after the operation and changed base on the results of the bacterial culture for at least 6 weeks.

All included patients were followed up regularly. Complications during bone transport and after the docking were recorded and managed accordingly. Patients were asked to obtained imaging test for regular assessment. When the transported segment reached the docking site and radiological examination established bridging callus appeared, the external fixator was removed after one month of dynamization. A plaster cast was applied after removal of external fixation for 4–6 weeks.

EFI was recorded as the ratio of days of external devices was assembled (d) to the length of the regenerated bone (cm) [23]. Using the criteria of Fischgrund [23], radiographs indicates that three complete cortices had formed in the regenerate and the docking site was healing, to evaluate the regenerate consolidation and healing of the docking union and record them. The bone and functional results were assessed according to ASAMI classification [1] and complications were classified according to Paley classification [24].

## Results

### General results

There were 18 patients include 12 male and 6 female with an average age of 40.4 years (ranged 22–62 years) were successfully followed up at minimum of 2 years after removal of external fixator. The details demographics of the patients are shown in Table 1. Mean tibial defect after radical debridement was 6.8 cm (ranged 6.0–8.2 cm) which were located proximal in 6 cases, middle in 8 cases and distal in 4 cases. The etiology of tibial defect includes posttraumatic osteomyelitis in 13 cases and primary osteomyelitis in 5 cases. An average of previous operation time was 3.4 times. All 18 patients were treated with unilateral external fixation frame. An average interval duration from the formation of induced membrane to trifocal bone transport was 4.8 weeks (ranged 4–7 weeks). An average time of follow-up after removal of external fixator was 28.5 month without recurrence of osteomyelitis. An average of EFI was 37.1 days/cm (range, 30.8–45.6 days/cm) and regenerate consolidation occurred at a mean of 124.7 days (ranged 105–153 days). Mean duration of docking union was 186.4 days (ranged 143–261 days).

**Table 1:** Demographic data of 18 patients

Case	Age/gender	Defect size (cm)	Defect site	Etiology of defect	Previous operation time	Duration of spacer (weeks)	Follow-up period (months)	EFI (days/cm)	Duration of regenerate consolidation (days)	Duration of docking union (days)
1	26/M	7.1	Distal	PO	2	6	25	41.6	107	261
2	55/M	6.8	Middle	PTO	5	4	36	33.2	112	143
3	33/M	6.1	Proximal	PO	2	4	25	34.5	145	167
4	41/F	6.5	Proximal	PTO	4	5	38	33.9	117	213
5	53/M	7.5	Distal	PTO	5	6	24	32.7	153	172
6	40/M	6.8	Middle	PTO	2	7	26	37.1	124	147
7	37/M	6.1	Middle	PTO	4	4	30	43.1	116	178
8	22/F	7.2	Proximal	PO	2	5	41	33.6	106	223
9	62/M	6.2	Middle	PTO	5	4	24	40.7	133	151
10	58/M	7.3	Middle	PTO	5	5	25	43.5	121	182
11	29/F	6.3	Distal	PO	1	4	24	36.1	113	194
12	35/M	6.0	Middle	PO	3	4	24	39.1	127	156
13	33/F	7.9	Proximal	PTO	3	6	27	37.6	131	238
14	45/M	8.2	Proximal	PTO	4	5	26	45.6	114	178
15	37/F	6.3	Middle	PTO	2	5	25	34.6	105	167
16	43/M	8.0	Distal	PTO	3	4	39	35.3	148	189
17	39/M	6.1	Middle	PTO	5	4	26	30.8	137	203
18	40/F	6.7	Proximal	PTO	3	5	29	34.7	136	193

*EFI* external fixation index, *M* male, *F* female, *PO *primary osteomyelitis, *PTO* posttraumatic osteomyelitis

### ASAMI score

**Fig. 1:**
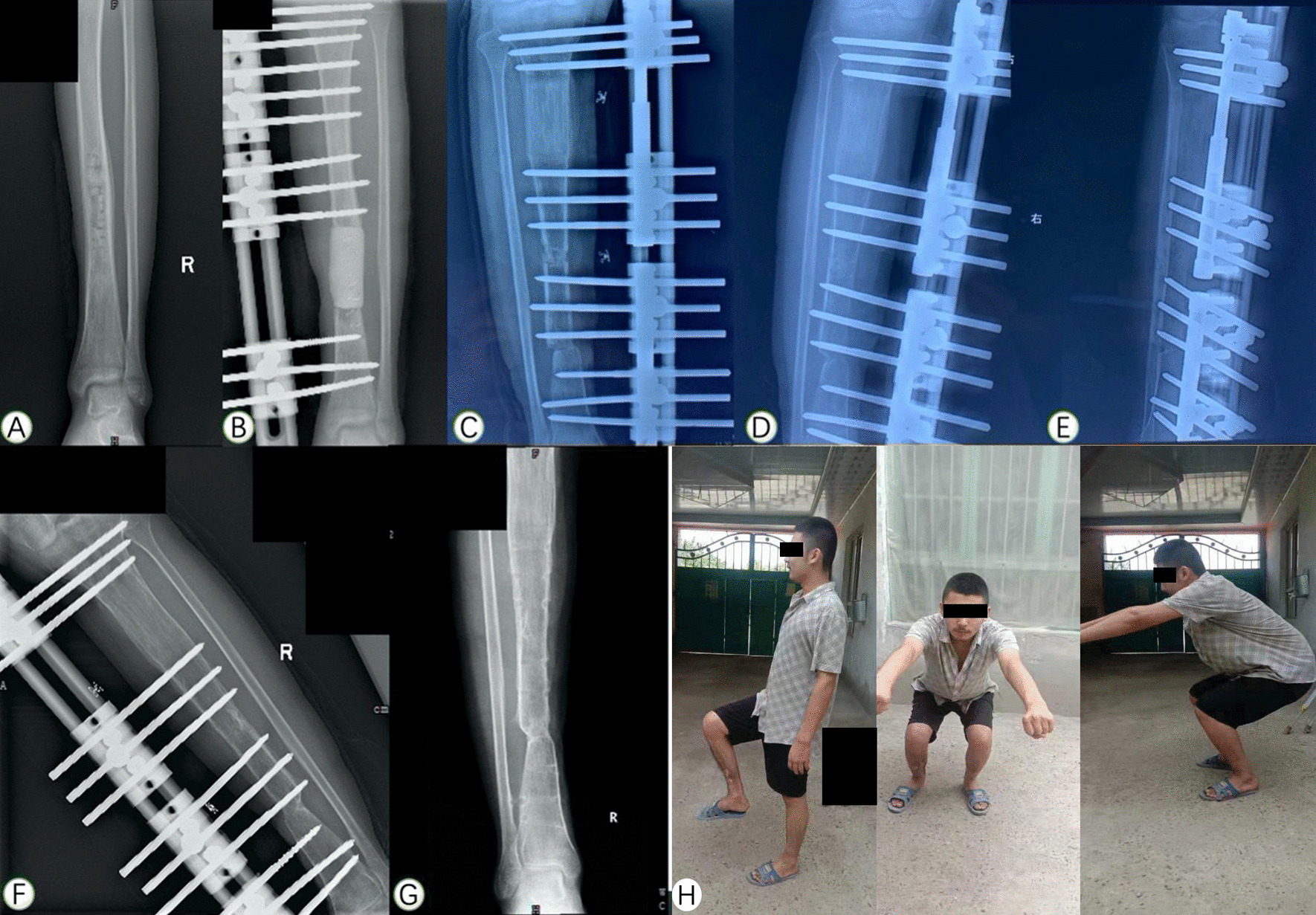
**A** An 43-year-old male patient with posttraumatic osteomyelitis of the right tibia. **B**–**E** After three previous debridement operations, there was a defect of 8.0 cm and filled with PMMA spacer. The soft tissue defect was covered by latissimus dorsi flap. **F** Two months after bifocal bone transport using monolateral external fixator. **G**, **H** External fixator was removed with excellent bone result assessed by ASAMI system 3 years after removal of external fixator. **I**–**K** Radiograph and general appearance at last visit with excellent functional result

**Fig. 2:**
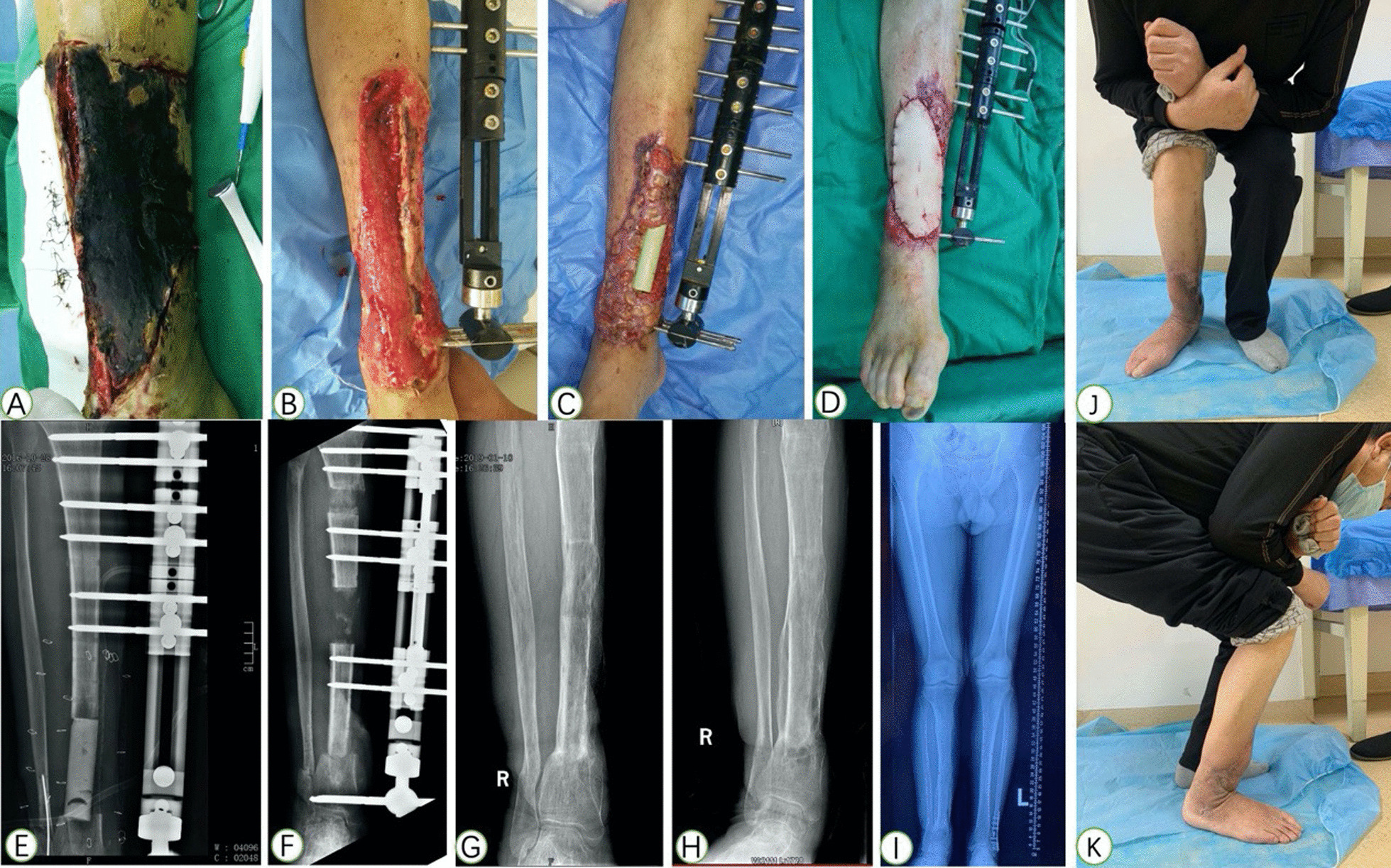
**A** An 26-year-old male patient with primary osteomyelitis of the right tibia. **B** An excision of infected bone with 7.1 cm defect and filled with cement spacer. **C**–**F** Trifocal bone transport was completed with good regenerate consolidation and docking union was achieved at 8 months after index surgery. **G** External fixator was removed with excellent bone result assessed by ASAMI system. **H**, **I** General appearance at last visit with excellent functional result

ASAMI criteria [1] was used to evaluate the bone union and functional recovery and shown in Table 2. The bony outcome was excellent in 6 patients, good in 8 patients, fair in 3 patients and poor in 1patient, and functional outcome was excellent in 4 patients, good in 10 patients, fair in 2 patients and poor in 2 patients (Figs. 1, 2).

**Table 2:** Evaluation of the bone and functional results according ASAMI classification

Outcomes	Numbers/percentage			
	Excellent	Good	Fair	Poor
Bone results	6 (33.3%)	8 (44.4%)	3 (16.7%)	1 (5.6%)
Functional results	4 (22.2%)	10 (55.6%)	2 (11.1%)	2 (11.1%)

*ASAMI* the Study and Application of the Method of Ilizarov

### Complications

The criteria recommended by Paley [24] were adopted to evaluate the complications which was demonstrated in Table 3. Muscle contraction was occurred in 11 cases and resolved by physiotherapy or subcutaneous tendon lengthening. Axial deviation occurred in 7 patients which was resolved by adjusting the frame or re-installing additional Schanz screws. Delayed consolidation and docking union occurred in 2 patients and docking site nonunion in 4 patients, which were successfully treated by using accordion maneuver or supplementary iliac bone grafting. The most common complication was pin tract infection which occurred in 15 cases, which was managed by regular pin tract care and oral antibiotics according to the bacterial culture result. Nine patients suffered knee or ankle joint (s) stiffness, which were treated by early physiotherapy and extending apparatus. There were no major complications such as nerve or vascular injury in the present study.

**Table 3:** Complications according Paley criteria

Parameter	Problems	Obstacles	Complications	Total
Muscle contraction	6	2	3	11
Axial deviation	0	3	4	7
Delayed consolidation	2	4	0	6
Pin problems	12	3	0	15
Repeat fracture	0	0	0	0
Joint stiffness	2	1	6	9
Other	1	3	0	4
Total	23	16	13	

## Discussion

Reconstruction of massive tibial defects caused by primary or secondary osteomyelitis is attracting considerable critical attention which require individualized strategic reconstructive approach to reach expected clinical outcomes [1]. Strategies of reconstruction have been proposed such as autologous bone grafting, Masquelet technique (also known as induced membrane technique), Ilizarov technique based on distraction osteogenesis, free vascularized or non-vascularized fibular grafting, depending on the anatomical location and size of the defect as well as associated injuries [2–10].

Masquelet technique is an evolving approach that is separated into two stages. A complete debridement is performed in the first stage to improve the local blood supply and remove the infected lesion. Following debridement, a PMMA spacer is inserted over the defect location. In a rebuilt post-infection environment, PMMA spacers provide various possible advantages. The presence of the PMMA cement leads to the body reaction encourages the formation of a biological membrane which exhibits the most abundant vascularization and possesses inductive properties for bone repair. Animal and human research have lately proven the effects of induced membranes on the enhancement of bone growth [14, 25]. In the second stage, the PMMA spacer will be removed and replaced by autologous or allogeneic bone graft. Alternatives after the removal of PMMA spacer include bone transport, titanium cages and mega prostheses [11, 25].

In contrast to the traditional Ilizarov technique, the placement of spacers solves problems during bone transport by preventing soft tissue insertion so that the bone segment can be transported in a stable environment after spacer was retrieved [12]. Previous publications have given grounds for the use of PMMA bone cement combined with a 10% vancomycin [11–16, 25]. Bone cement mixed with antibiotics can be released several weeks after insertion at the infection site. Local antibiotic concentrations are approximately 200 times higher than systemic dosing, and also prevent bacterial development and produce a relatively sterile environment. Studies have demonstrated that antibiotic elution times ranged from 1 week to 6 months from PMMA spacer [26, 27]. Another research established a strong link between antibiotic elution and the amount of antibiotic added to the PMMA spacer [28].

The best period for PMMA spacer duration employing an induced membrane reconstruction has yet to be identified definitely. The induced membrane is a rich source of mesenchymal stem cells, with mature vascularized fibrous tissue that may release a range of growth factors, and its osteogenic and neovascularization activity peak 2–4 weeks after development and gradually decline after 6 weeks [13, 29]. As a result, the next round of treatment should begin within 1 month. In this study, the average interval duration from the formation of induced membrane to trifocal bone transport was 4.8 weeks.

Four patients in this study had concomitant soft tissue defects, two of which were treated with latissimus dorsi flap transfer in the first stage. The other two patients had small wounds that healed during bone transport. We believe that the early application of the flap for larger wounds can reduce the chance of reinfection and decrease the number of dressing changes. Reducing the pain and financial burden of the patient. For smaller wounds, the skin and soft tissues can be transported simultaneously during bone transport, covering the wound for a shorter period of time, without flap transfer. Ilizarov bone transport technique based on distraction osteogenesis have been applied in treatment of bone defect, especially in cases associated with soft tissue loss given its naturally advantages of simultaneous histogenesis result in expected treatment outcomes. The main drawback of this technique is the long duration of the external fixation, which causes many life and psychological obstacles to the patient.

Psychological disorders such as interpersonal sensitivity, sleep disturbances, obsessive–compulsive symptoms and anxiety have been proposed in patients treated with external fixations for more than 8 months [30]. To overcome the inevitable long duration of external fixation time using bifocal bone transport technique and reduce its associated complications, multifocal transport has been conducted to reduce the time spent on external fixator and its associated complications. Catagni et al. [21] suggested that double-level bone transport can significantly shorten the treatment time in the reconstruction of tibial defect, and reduce the number of additional operations and related complications. Yushan et al. [31] indicated that compared with bifocal bone transport approach, it might considerably lower the lengthening index with superior functional outcomes by trifocal bone transport. Borzunov et al. [1] also concluded that the bone lengthening time and the consolidation time in multi-level group can be reduced by 2.5 times and 1.3–1.9 times. Several investigations have been conducted and discussed on the application of cell preparations such as transforming growth factor (TGF), fibroblast growth factor (FGF), insulin-like growth factor (IGF), bone morphogenic protein (BMP), and bone marrow aspirate concentrate (BMAC) to advance osteogenesis, bone consolidation, and then further reduce prolonged external fixation [32–35]. Furthermore, angiogenesis is crucial in enhancing bone regeneration. Researches have indicated that vascular endothelial growth factor (VEGF), platelet-derived growth factor (PDGF) and angiopoietin are essential for the formation of neovascularization during distraction osteogenesis, which can promote bone consolidation and enhance rapid osseointegration [36, 37]. Additionally, adjunctive therapy such as the use of platelet rich plasma (PRP) or hyperbaric oxygen therapy (HOT), local injection of teriparatide in the treatment of septic or aseptic tibial nonunion while combined with Ilizarov technique, all of which have demonstrated that benefited to the docking union, accelerate rapid healing of the regenerated bone and an early removal of external fixator [38, 39].

The need for early soft tissue covering in the treatment of massive bone defects with soft tissue defects is still a point of contention. Spiegl et al. [16] indicated that wounds should have early soft tissue coverings to reduce dead space, restore appropriate local blood supply to maximize immunocompetence, and boost local antibiotic concentrations. Peng et al. [15] proposed using a negative pressure wound dressing approach to temporarily close the wound while waiting for granulation tissue to cover the incision and then implanting skin to entirely repair the wound. Slow skin traction is used to close wounds that have insufficient granulation tissue production. A recent study employed the Ilizarov approach to repair massive tibial and soft tissue defects by stretching the skin and subcutaneous tissue with the pins and screws of the Ilizarov ring. Before the bone ends made contact at the docking location, the soft tissue defect was covered with newly formed soft tissue [40].

Combination use of antibiotic cement spacers and bone transport (bifocal) technique have been reported in recent years. Van Niekerk et al. [13] compared the outcome of combined circular external fixation and PMMA spacer application between patients with open fractures and infected tibial nonunions and concomitant segmental bone loss. The mean EFI for both groups was 46.9 ± 22.4 days/cm. Marais et al. [12] treated 7 patients with circular external fixation and cement spacer, and the mean EFI was 81 days/cm. Spiegl et al. have published a series of cases with chronic osteomyelitis and reported an average EFI of 57 days/cm [16]. In this study, an average of EFI was 37.1 days/cm (ranged 30.8–45.6 days/cm). In a previous study, which compared bifocal and trifocal (double-level) bone transport using traditional Ilizarov methods, the external fixation index was 32.9 ± 9.21 days/cm in TF group, and 62.21 ± 24.60 days/cm in BF group [31]. There were a few other published studies that discussed using the Ilizarov bone transport technique to treat massive tibial defects caused by osteomyelitis, which were summarized in Table 4 for comparison [11, 21, 41–51]. The differences in EFI can be ascribed to the etiology of the osteomyelitis as well as the size of the bone defect. Using PMMA spacers necessitates a longer period of external fixation. The period spent waiting for the induce membrane to develop before bone transport begins might partly explain this.

**Table 4:** Summary of study and patient characteristics

Study	Study type	Patients (M/F)	Mean ages (years)	Mean defect sizes (cm, range)	Treatment type (trifocal/bifocal)	Bone union rate	Bony results (excellent/good/fair/poor)	Functional results (excellent/good/fair/poor)	EFI (days/cm)	Follow-up (months)
Abuomira et al. [41]	IBT	55 (44/11)	41.5	7.1 (3–17)	29/26	89%	28/18/5/4	25/21/5/4	nr	50
Bernstein et al. [42]	IBT	30 (24/6)	43	5.7 (1.6–12)	0/30	77%	27/2/0/1	27/1/0/0	nr	33
Meleppuram et al. [43]	IBT	42 (32/10)	38	nr (2.5–5.5)	nr/36	100%	25/6/11/0	23/13/2/4	nr	14
Kinik et al. [44]	IBT	30 (28/2)	39.5	8.1 (6–15)	0/30	96.66%	22/6/0/0	19/7/2/0	44.7	32.5
Fahad et al. [45]	IBT	51 (41/10)	45.7	3.5 (2–5)	0/51	96%	22/19/7/3	24/21/4/2	60	36.8
Rohilla et al. [46]	IBT	70 (62/8)	31.3	5.8 (3–9) vs 5.8 (3–10)	0/70	77% vs 80%	35/27/3/5	38/27/1/3	63.6 vs 63.3	33.8 vs 32.6
Catagni et al. [21]	IBT	86 (77/9)	42 vs 43	13.5 (10.5–16.5) vs 12.5 (9.6–14.4)	41/45	100%	68/11/3/4	47/21/14/4	41/44	nr
Zhang et al. [47]	IBT	16 (9/7)	39.1	10.9 (6–20)	16/0	100%	10/0/6/0	12/4/0/0	33	29.5
Li et al. [48]	IBT	26 (20/6)	40.4	10.7 (7.5–15) vs 7.2 (5.8–9)	13/13	100%	20/0/6/0	22/4/0/0	36.6 vs 75.6	28.5
Paley et al. [49]	IBT	19 (14/5)	38	10.7 (2–20)	6/13	100%	15/3/1/0 (paley)	12/6/1/0 (paley)	51	78
Tone et al. [50]	IM	20 (15/5)	39.9	6.69	–	100%	5/10/4/1	8/9/3/0	54.9	23.2
Morris et al. [11]	IM	12 (9/3)	35	5.8 (2–15)	–	42%	nr	nr	nr	22.5
El-Alfy et al. [51]	IM	15 (12/3)	32	8 (5–14)	–	87%	nr	nr	nr	23
Present study	IM + IBT	18 (12/6)	40.4	6.8 (6–8.2)	18/0	100%	6/8/3/1	4/10/2/2	37.1	28.5

*EFI* external fixation index, *M* male, *F* female, *IBT* Ilizarov bone transport, *IM* induced membrane, *nr* not reported

In our series, there was a significant rate of complications. Pin site infection was the most typical complication, occurring in 15 instances, which has a high incidence in previous studies [29]. Improvements to the procedure of pin tract care and the therapy of systemic antibiotics were beneficial. Muscle contraction was caused by muscular tension, leading to distraction and strength imbalance between the flexors and extender. Physiotherapy or subcutaneous tendon lengthening. Nine patients suffered knee or ankle joint (s) stiffness, which were successfully treated by early physiotherapy and extending apparatus.

The authors acknowledge that there are limitations to this study. First, this study was designed as a single-center retrospective study which was subject to selection and indication biases. Further prospective studies are needed in the future to address methodological limitations. Second, there is only one cohort with 18 patients and no control group. Although a randomized, multicenter controlled trial would be ideal, such a trial would encounter ethical difficulties.

## Conclusion

Combination use of induced membrane followed by trifocal bone transport technique in the reconstruction of massive tibial defect caused by osteomyelitis is an effective treatment approach. While the procedure does not seem to offer an advantage in regard to the external fixation index and complications. It might be a valuable addition to the infection resolution. Further clinical studies with longer follow-up period are necessary.

## Data Availability

All data generated or analyzed during this study are included in this published article.
